# Predicting Alzheimer's disease development: a comparison of cognitive criteria and associated neuroimaging biomarkers

**DOI:** 10.1186/s13195-015-0152-z

**Published:** 2015-11-05

**Authors:** Brandy L. Callahan, Joel Ramirez, Courtney Berezuk, Simon Duchesne, Sandra E. Black, for the Alzheimer’s Disease Neuroimaging Initiative

**Affiliations:** LC Campbell Cognitive Neurology Research Unit, Sunnybrook Health Sciences Centre, 2075 Bayview Avenue, Rm A4 21, Toronto, Ontario M4N 3 M5 Canada; Heart & Stroke Foundation Canadian Partnership in Stroke Recovery, Sunnybrook Health Sciences Centre, Toronto, Canada; Sunnybrook Health Sciences Centre, Brain Sciences Research Program, Sunnybrook Research Institute, Toronto, Canada; Université Laval, Faculté de médecine (Radiologie), Québec, Canada; Centre de recherche de l’Institut universitaire en santé mentale de Québec, Québec, Canada; Department of Medicine (Neurology), University of Toronto, Institute of Medical Science, Québec, Canada

## Abstract

**Introduction:**

The definition of “objective cognitive impairment” in current criteria for mild cognitive impairment (MCI) varies considerably between research groups and clinics. This study aims to compare different methods of defining memory impairment to improve prediction models for the development of Alzheimer’s disease (AD) from baseline to 24 months.

**Methods:**

The sensitivity and specificity of six methods of defining episodic memory impairment (< −1, −1.5 or −2 standard deviations [SD] on one or two memory tests) were compared in 494 non-demented seniors from the Alzheimer’s Disease Neuroimaging Initiative using the area under the curve (AUC) for receiver operating characteristic analysis. The added value of non-memory measures (language and executive function) and biomarkers (hippocampal and white-matter hyperintensity volume, brain parenchymal fraction [BPF], and APOEε4 status) was investigated using logistic regression.

**Results:**

Baseline scores < −1 SD on two memory tests predicted AD with 75.91 % accuracy (AUC = 0.80). Only APOE ε4 status further improved prediction (B = 1.10, SE = 0.45, *p* = .016). A < −1.5 SD cut-off on one test had 66.60 % accuracy (AUC = 0.77). Prediction was further improved using Trails B/A ratio (B = 0.27, SE = 0.13, *p* = .033), BPF (B = −15.97, SE = 7.58, *p* = .035), and APOEε4 status (B = 1.08, SE = 0.45, *p* = .017). A cut-off of < −2 SD on one memory test (AUC = 0.77, SE = 0.03, 95 % CI 0.72-0.82) had 76.52 % accuracy in predicting AD. Trails B/A ratio (B = 0.31, SE = 0.13, *p* = .017) and APOE ε4 status (B = 1.07, SE = 0.46, *p* = .019) improved predictive accuracy.

**Conclusions:**

Episodic memory impairment in MCI should be defined as scores < −1 SD below normative references on at least two measures. Clinicians or researchers who administer a single test should opt for a more stringent cut-off and collect and analyze whole-brain volume. When feasible, ascertaining APOE ε4 status can further improve prediction.

## Introduction

Patients with mild cognitive impairment due to Alzheimer’s disease (MCI) [1] - also known as mild neurocognitive disorder [2] - are considered to be at an early stage of dementia. There are now multiple published criteria sets for identifying these individuals at high risk of progression [1–3], all of which include at least: 1) subjective concern; 2) an objective cognitive impairment on formal neuropsychological testing in one or more cognitive domains, typically including memory; 3) preservation of functional independence; and 4) no dementia.

Although these criteria have been a major step forward in the conceptualization of MCI, they leave room for considerable ambiguity, particularly regarding the operational definition of objective cognitive impairment. A number of cognitive tests have been proposed that may be useful for identifying objective episodic memory impairment in MCI, specifically measures that assess both immediate and delayed recall, such as word-list learning or paragraph recall [1, 4]. These suggestions are very useful in providing common ground for clinicians and researchers working with MCI cohorts. However, three critical issues remain.

First, it is unclear which cutoff scores should be used to define impairment. Studies examining MCI patients typically report test performance in the range of one to two standard deviations (SD) below age-adjusted and/or education-adjusted norms. However, using a −1 SD cutoff may be overly inclusive, as cognitive performance in healthy older adults often falls below this limit [5] for a variety of non-pathological reasons (e.g., fatigue, anxiety). Conversely, using a −2 SD cutoff may underestimate the number of individuals who are in the earliest phases of the disease process.

Second, it is unclear how many measures should be used in assessing cognition. In memory clinics, diagnosis is typically based on results of a battery of neuropsychological tests including more than one test probing the same cognitive domain. Longitudinal evidence confirms that using at least two tests to establish impairment greatly increases diagnostic accuracy [6]. In research settings, however, MCI diagnosis is often based on a single test. This is potentially problematic, as research has shown that more than one quarter of healthy elderly adults who are tested using a single memory measure obtain scores in impaired ranges (< −1.5 SD), while this number is reduced to 14.1 % when a second test is added [5]. As mentioned above, impaired performance on a single test in otherwise healthy normal adults may be explained by numerous factors such as anxiety, depression, fatigue, or inattention. Thus, this single-test procedure may not be adequate for identifying individuals who are at highest risk of dementia.

Third, it is unclear which cognitive domain(s) should be assessed, if any, in addition to episodic memory. Originally, Petersen’s [3] diagnostic criteria recommended that a distinction be made between single-domain and multiple-domain MCI, with the assumption that this classification would be of heuristic value in determining the probable etiology of the disorder. This recommendation is echoed in Albert and colleagues’ [1] revised criteria as well. Indeed, some longitudinal evidence suggests that these subtypes evolve differently over time [7], suggesting distinct etiological processes. However, the most recent DSM-5 criteria for mild neurocognitive disorder [2] do not discriminate between single-domain and multiple-domain cognitive impairment. Many research studies also do not make this distinction.

In addition, recent guidelines for diagnosing MCI have emphasized the importance of using genetic and imaging biomarkers in addition to neuropsychological testing. The presence of one or two copies of the epsilon 4 allele (ε4) in the apolipoprotein E (*APOE*) gene is one commonly accepted genetic characteristic believed to increase the risk of development of dementia due to Alzheimer’s disease (AD) [8]. Additionally, metrics obtained from structural magnetic resonance imaging (MRI) that assess neuronal injury, such as total brain atrophy [9, 10], ventricular enlargement [11–13], hippocampal (HP) volume loss [14, 15], medial temporal lobe atrophy [16], and possibly the presence of small vessel disease [17], may be informative predictors for the development of AD dementia.

Using data obtained from the Alzheimer’s Disease Neuroimaging Initiative (ADNI), the purpose of this study is to determine whether prediction of development of clinical dementia among non-demented participants is improved by: 1) using cutoff scores of −1.0, −1.5 or −2.0 SD to define cognitive impairment; 2) assessing episodic memory using one or two tests; 3) assessing additional non-memory domains; and 4) accounting for commonly used neuroimaging and genetic biomarker data. It was hypothesized that the identification of individuals at risk for the development of dementia would best be predicted by defining objective impairment as performance < −1 SD on two episodic memory tests. Furthermore, it was anticipated that the ability to predict the development of AD would be further optimized by considering performance in at least one other, non-memory domain. Finally, it was expected that the inclusion of imaging and genetic biomarkers known to be associated with AD would further improve prediction.

## Materials and methods

Data used in the preparation of this article were obtained from the ADNI database (adni.loni.usc.edu) on 3 February 2015. The ADNI was launched in 2003 as a public–private partnership, led by Principal Investigator Michael W. Weiner, MD. The primary goal of ADNI has been to test whether serial MRI, positron emission tomography (PET), other biological markers, and clinical and neuropsychological assessment can be combined to measure the progression of MCI and early AD.

### Participants

Of the 819 participants enrolled in ADNI-1, those who had neuropsychological and genetic data available at baseline and 24-month follow up were selected for this study (n = 630). A 24-month follow-up period was selected to maximize statistical power and to ensure that harmonized imaging outcome measures were available for the majority of the sample. Of these 819 participants, those with a diagnosis of probable AD at baseline were excluded (n = 136). Individuals with a history of neurological or psychiatric illness or substance abuse, or without a study partner able to corroborate reports of functioning, were not eligible for ADNI; complete eligibility criteria for the ADNI study as a whole are described at http://adni.loni.usc.edu/wp-content/uploads/2010/09/ADNI_GeneralProceduresManual.pdf. The final sample consisted of the remaining 494 non-demented participants. According to the assigned diagnoses in the ADNI database, 294 of these participants were classified as having MCI, and the remaining 200 were classified as cognitively normal. All participants (201 women, 293 men) were 55–89 years old at baseline (mean = 75.3 ± 6.4) and had 6–20 years of education (mean = 15.9 ± 2.8).

### Measures

#### Cognitive measures

A neuropsychological battery was administered to all participants upon admission to ADNI, and raw scores were downloaded from the ADNI Neuropsychological Battery table. Of interest in the present study are tests that measure general cognition (Mini-mental state exam (MMSE)), episodic memory (Logical memory story A delayed recall (LM-II), Rey auditory verbal learning test (AVLT)), language (Category fluency, Boston naming test (BNT)) and executive functioning (Trails A and B). A derived Trails B/Trails A ratio was calculated to obtain a relatively independent measure of executive control, as has been suggested by other authors [18]. Raw scores were transformed to standardized scores (*z* scores or scaled scores (SS)) based on published age-adjusted norms for the AVLT [19], Category fluency [20], BNT [21] and Trails A & B [18]. Education-adjusted *z* scores for LM-II story A were obtained using a web-based calculator [22] based on data from a large published report [23]. Higher *z* scores or SS represent better performance, with the exception of Trails A and B in which higher scores represent poorer performance (i.e., longer time to complete the test).

### Outcome measure

The presence or absence of clinically probable AD was assessed at 24 months and defined as: 1) MMSE <26; 2) Clinical Dementia Rating (CDR) ≥0.5; and 3) positive NINCDS/ADRDA criteria for probable AD [24].

### Imaging and genetic biomarkers

Neuroimaging-based biomarkers were obtained from downloaded ADNI database tables (hierarchical parcellation of MRI using multi-atlas labeling methods (UPENN); white matter hyperintensity volumes (UCD)). Whole brain atrophy was assessed using the brain parenchymal fraction (BPF), which was calculated as a ratio of total parenchymal volume (gray matter (GM) and white matter (WM)) to total cranial vault (TCV) volume as follows:

$$ \mathrm{B}\mathrm{P}\mathrm{F} = \left(\mathrm{G}\mathrm{M} + \mathrm{W}\mathrm{M}\right)/\mathrm{T}\mathrm{C}\mathrm{V}. $$

To assess medial and focal atrophy, head-size-corrected ventricular cerebrospinal fluid (vCSF) and HP volume were automatically segmented using previously published and validated methods [11, 14]. Small vessel disease burden was assessed using whole brain white matter hyperintensity (WMH) volumes [25]. Full segmentation methodological details can be obtained from ADNI (see ADNI1_Methods_UCD_WMH_Volumes_Methods.pdf and ADNI_Total_Cranial_Vault_Segmentation_Method_20121108.pdf). In addition, the presence of one or two copies of the APOE ε4 allele was determined for all participants as per standard ADNI protocol.

### Statistical analyses

Six binary variables were created based on scores < −1.0, −1.5 or −2.0 SD on one (LM-II or AVLT delayed recall) or two (LM-II and AVLT delayed recall) memory tests, and participants were classified as above or below each cutoff. The predictive accuracy of these six cutoffs was tested using the area under the curve (AUC) for receiver operating characteristic (ROC) analysis. The minimum value for an AUC to be considered clinically significant was >0.75 [26]. Hanley and McNeil’s [27] method was used to test for statistical differences between AUC values. Cutoff scores with AUC values >0.75 were then entered into separate binary logistic regression analyses with hierarchical designs, with probable AD at 24 months as the binary (yes/no) dependent variable. In all models, age, sex, education, MMSE and the selected cutoff score were entered in a first block. A second block included performance on non-memory cognitive measures, specifically standardized Category fluency, BNT, and Trails B/A- derived scores. A third block assessed the potential added predictive value of biomarkers that are known to be associated with probable AD: BPF, vCSF volume, total HP volume, WMH volume, and APOE ε4 status. We verified that all variables met multicollinearity and linearity assumptions.

Last, in order see whether participants whose performance fell above and below the best selected cutoff scores were phenotypically different, multivariate analysis of covariance (MANCOVA) was used to compare cognitive and neuroimaging characteristics between these two groups, with age, sex and education entered as covariates. Highly skewed variables exhibiting non-normal distributions were log-transformed (WMH, vCSF) or inverse-transformed (Trails B/A ratio) prior to analysis. Category fluency scores did not meet the equal variance assumption and were therefore log-transformed. Dichotomous variables were compared using the chi-square test.

## Results

**Fig. 1:**
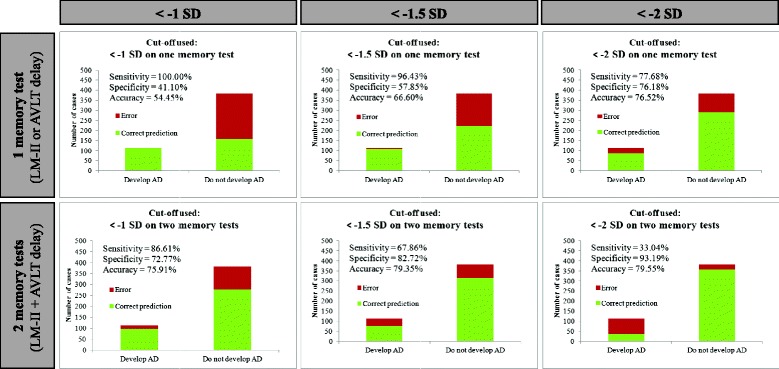
Sensitivity, specificity and accuracy of different cutoff scores in 494 non-demented participants at baseline. *AD* Alzheimer’s disease, *LM-II* Logical memory story A delayed recall, *AVLT* Rey auditory verbal learning test

At 24 months post-baseline, 112 participants (22.7 %) had received a diagnosis of AD. Sensitivity, specificity and accuracy of the different cutoff scores are illustrated in Fig. 1. On ROC analysis there were three cutoffs with AUC values >0.75. A cutoff of < −1 SD on two memory tests (AUC = 0.80, standard error (SE) = 0.02, 95 % CI 0.75, 0.84) had 75.91 % accuracy in correctly identifying patients who would later develop probable AD (97 true positives) and those who would not (278 true negatives). A cutoff of < −1.5 SD on one memory test (AUC = 0.77, SE = 0.02, 95 % CI 0.73, 0.81) had 66.60 % accuracy (108 true positives, 221 true negatives). A cutoff of < −2 SD on one memory test (AUC = 0.77, SE = 0.03, 95 % CI 0.72, 0.82) had 76.52 % accuracy (87 true positives, 291 true negatives). The AUC values for the three cutoff scores were not statistically different (all comparisons *p* >0.05, one-tailed).

Seven participants were excluded from subsequent analyses because they had missing data (two had missing WMH data, two had missing Trails B data, one had missing BNT data, and two had missing Trails B and BNT data). First, on logistic regression model to test the added value of non-memory measures and biomarkers, in addition to a cutoff of < −1 SD on two memory tests (*B* = 2.55, SE = 0.33, *p* <0.001), MMSE was a significant predictor of future AD (*B* = −0.34, SE = 0.08, *p* <0.001). Only the presence of two APOE ε4-positive alleles (*B* = 1.10, SE = 0.45, *p* = 0.016) further improved prediction. Altogether, this model accounted for 83.4 % of the variance in risk of probable AD (Table 1).

**Table 1:** Variables predicting AD in addition to < −1 SD on two episodic memory tests

		Block by block			Final model		
Predictor	Nagelkerke’s *R* ^*2*^	Odds ratio	Confidence interval	*P*	Odds ratio	Confidence interval	*P*
Block 1: main predictors	0.422						
Age		1.031	1.0, 1.1	0.146	1.006	1.0, 1.1	0.812
Education		0.987	0.9, 1.1	0.780	0.989	0.9, 1.1	0.830
Sex		1.131	0.7, 2.0	0.662	1.163	0.6, 2.1	0.626
Mini-mental state exam		0.712	0.6, 0.8	<0.001	0.763	0.6, 0.9	0.001
< −1 SD on two tests		12.845	6.7, 24.6	<0.001	8.082	3.9, 16.8	<0.001
Block 2: non-memory tests	0.439						
Category fluency		0.853	0.7, 1.1	0.227	0.869	0.7, 1.1	0.314
Boston naming test		0.944	0.9, 1.0	0.167	0.948	0.9, 1.0	0.227
Trails B/A ratio		1.215	0.9, 1.6	0.130	1.142	0.9, 1.5	0.342
Block 3: biomarkers	0.490						
Apolipoprotein E ε4							0.031
Apolipoprotein E ε4 (1 allele)					1.797	1.0, 3.3	0.055
Apolipoprotein E ε4 (2 alleles)					2.992	1.2, 7.3	0.016
Total hippocampal volume					0.081	0.0, 12.3	0.326
Brain parenchymal fraction					0.000	0.0, 27.9	0.123
White matter hyperintensity					1.085	1.0, 1.2	0.121
Ventricular cerebrospinal fluid					1.135	0.9, 1.4	0.240

In the second model, in addition to a cutoff of < −1.5 SD on one memory test (*B* = 3.09, SE = 0.54, *p* <0.001), significant predictors of probable AD were MMSE (*B* = −0.32, SE = 0.07, *p* <0.001) and the Trails B/A ratio in the non-memory cognitive measures block (*B* = 0.27, SE = 0.13, *p* = 0.033). Biomarkers that significantly improved prediction included BPF (B = −16.58, SE = 7.64, *p* = 0.030) and presence of two APOE ε4-positive alleles (B = 1.05, SE = 0.45, *p* = 0.021). This model accounted for 82.3 % of the variance in risk of probable AD (Table 2).

**Table 2:** Variables predicting AD in addition to < −1.5 SD on one episodic memory test

		Block by block			Final model		
Predictor	Nagelkerke’s *R* ^*2*^	Odds ratio	Confidence interval	*P*	Odds ratio	Confidence interval	*P*
Block 1: main predictors	.396						
Age		1.000	1.0, 1.0	0.997	0.982	0.9, 1.0	0.454
Education		0.993	0.9, 1.1	0.870	0.994	0.9, 1.1	0.893
Sex		1.028	0.6, 1.7	0.917	1.025	0.6, 1.8	0.935
Mini-mental state exam		0.729	0.6, 0.8	<0.001	0.811	0.7, 1.0	0.010
< −1.5 SD on one test		22.061	7.7, 63.0	<0.001	12.390	4.2, 36.8	<0.001
Block 2: non-memory tests	.425						
Category fluency		0.834	0.6, 1.1	0.163	0.854	0.7, 1.1	0.252
Boston naming test		0.937	0.9, 1.0	0.109	0.937	0.9, 1.0	0.127
Trails B/A ratio		1.314	1.0, 1.7	0.033	1.216	0.9, 1.6	0.164
Block 3: biomarkers	.482						
Apolipoprotein E ε4							0.036
Apolipoprotein E ε4 (1 allele)					1.767	1.0, 3.2	0.055
Apolipoprotein E ε4 (2 alleles)					2.853	1.2, 7.0	0.021
Total hippocampal volume					0.015	0.0, 1.7	0.081
Brain parenchymal fraction					0.000	0.0, 0.2	0.030
White matter hyperintensity					1.025	0.9, 1.1	0.654
Ventricular cerebrospinal fluid					1.043	0.9, 1.3	0.687

In the third model, in addition to a cutoff of < −2 SD on one memory test (*B* = 2.04, SE = 0.28, *p* <0.001), significant predictors of probable AD were MMSE (*B* = −0.40, SE = 0.08, *p* <0.001) and the Trails B/A ratio in the non-memory cognitive measures block (*B* = 0.31, SE = 0.13, *p* = 0.017). Presence of two APOE ε4-positive alleles (*B* = 1.07, SE = 0.46, *p* = 0.019) further improved prediction. This model accounted for 81.9 % of the variance in risk of probable AD (Table 3).

**Table 3:** Variables predicting AD in addition to < −2 SD on one episodic memory test

		Block by block			Final model		
Predictor	Nagelkerke’s *R* ^*2*^	Odds ratio	Confidence interval	*p*	Odds ratio	Confidence interval	*p*
Block 1: main predictors	.380						
Age		1.017	1.0, 1.1	0.403	0.992	0.9, 1.0	0.735
Education		0.981	0.9, 1.1	0.668	0.992	0.9, 1.1	0.877
Sex		0.843	0.5, 1.4	0.528	0.868	0.5, 1.6	0.639
Mini-mental state exam		0.672	0.6, 0.8	<0.001	0.756	0.6, 0.9	0.001
< −2 SD on one test		7.652	4.4, 13.3	<0.001	4.722	2.6, 8.7	<0.001
Block 2: non-memory tests	.416						
Category fluency		0.850	0.7, 1.1	0.211	0.859	0.7, 1.1	0.268
Boston naming test		0.923	0.9, 1.0	0.052	0.925	0.9, 1.0	0.071
Trails B/A ratio		1.367	1.1, 1.8	0.017	1.275	1.0, 1.7	0.089
Block 3: biomarkers	.469						
Apolipoprotein E ε4							0.035
Apolipoprotein E ε4 (1 allele)					1.748	1.0, 3.1	0.060
Apolipoprotein E ε4 (2 alleles)					2.910	1.2, 7.1	0.019
Total hippocampal volume					0.018	0.0, 2.7	0.115
Brain parenchymal fraction					0.000	0.0, 4.7	0.078
White matter hyperintensity					1.029	0.9, 1.1	0.609
Ventricular cerebrospinal fluid					1.098	0.9, 1.4	0.382

Participants who scored above (n = 291) and below (n = 196) a cutoff score of < −1 SD on two memory tests were compared using MANCOVA. Levene’s test indicated that both groups had equal variances (all variables *p* >0.05). As summarized in Table 4, it was found that those with episodic memory scores below the cutoff had poorer performance on Category fluency (*F* (4,482) = 14.23, *p* <0.001), BNT (*F* (4,482) = 25.60, *p* <0.001), and Trails B/A ratio (*F* (4,482) = 7.18, *p* <0.001). For brain morphology, patients below the cutoff had smaller BPF (*F* (4,482) = 49.02, *p* <0.001), smaller left (*F* (4,482) = 44.83, *p* <0.001) and right HP volumes (*F* (4,482) = 41.03, *p* <0.001), more vCSF (*F* (4,482) = 28.99, *p* <0.001) and smaller WMH volume (*F* (4,482) = 8.69, *p* <0.001).

**Table 4:** Characteristics (mean (SD)) of participants above and below selected cutoffs

< −1.0 SD on two memory tests	Below cutoff	Above cutoff	*F*	*P*
	(n = 196)	(n = 291)		
Category fluency, *z* score	−0.40 (1.11)	0.38 (1.28)	14.23	<0.001
BNT, scaled score	10.09 (3.62)	12.55 (3.56)	25.60	<0.001
Trails B/A ratio, *z* score	0.01 (1.14)	−0.45 (0.82)	7.18	<0.001
BPF	0.81 (0.03)	0.82 (0.03)	49.02	<0.001
Left HP volume, cm^3^	0.23 (0.03)	0.27 (0.03)	44.83	<0.001
Right HP volume, cm^3^	0.25 (0.03)	0.28 (0.03)	41.03	<0.001
v CSF, cm^3^	3.52 (1.59)	3.19 (1.49)	28.99	<0.001
WMH, cm^3^	0.63 (1.34)	0.87 (2.70)	8.69	<0.0001
< −1.5 SD on one memory test	Below cutoff	Above cutoff	*F*	*P*
	(n = 264)	(n = 223)		
Category fluency, *z* score	−0.31 (1.11)	0.51 (1.31)	14.23	<0.001
BNT, scaled score	10.47 (3.71)	12.85 (3.45)	24.00	<0.001
Trails B/A ratio, *z* score	−0.11 (1.08)	−0.45 (0.83)	3.81	0.005
BPF	0.81 (0.03)	0.82 (0.02)	44.99	<0.001
Left HPV, cm^3^	0.24 (0.03)	0.27 (0.03)	27.38	<0.001
Right HPV (cm^3^)	0.25 (0.03)	0.29 (0.03)	33.42	<0.001
v CSF, cm^3^	3.53 (1.63)	3.07 (1.40)	28.94	<0.001
WMH, cm^3^	0.82 (2.31)	0.72 (2.19)	8.89	<0.001
< −2.0 SD on one memory test	Below cutoff	Above cutoff	*F*	*P*
	(n = 174)	(n = 313)		
Category fluency, *z* score	−0.42 (1.05)	0.33 (1.31)	11.61	<0.001
BNT, scaled score	10.19 (3.71)	12.32 (3.60)	19.22	<0.001
Trails B/A ratio, *z* score	−0.08 (1.12)	−0.37 (0.89)	3.40	0.009
BPF	0.81 (0.03)	0.82 (0.03)	45.07	<0.001
Left HPV, cm^3^	0.23 (0.03)	0.26 (0.03)	31.79	<0.001
Right HPV, cm^3^	0.25 (0.03)	0.28 (0.03)	35.16	<0.001
v CSF, cm^3^	3.52 (1.59)	3.22 (1.50)	28.72	<0.001
WMH, cm^3^	0.85 (2.64)	0.73 (2.01)	9.33	<0.001

For *z* scores, the mean is 0 and the standard deviation is 1. For scaled scores, the mean is 10 and the standard deviation is 3. Data are missing for seven participants. *BNT* Boston naming test, *BPF* brain parenchymal fraction, *HP* hippocampal volume, *vCSF* ventricular cerebrospinal fluid, *WMH* white matter hyperintensities

Participants who scored above (n = 223) and below (n = 264) a cutoff score of < −1.5 SD on one memory test were compared in a second MANCOVA. Two variables violated Levene’s test (Trails B/A ratio and left HP volume), likely due to the large sample sizes. Inspection of the data showed that the variance between both groups was highly similar (in the above-cutoff and below-cutoff groups, the respective variances were 0.010 and 0.016 for Trails B/A ratio, and 0.001 and 0.001 for left HP volume), and therefore parametric analyses were retained. Results revealed that individuals with episodic memory scores below the cutoff had poorer performance on Category fluency (*F* (4,482) = 14.24, *p* <0.001), BNT (*F* (4,482) = 24.00, *p* <0.001), and Trails B/A ratio (*F* (4,482) = 3.81, *p* = 0.005). They also had smaller BPF (*F* (4,482) = 45.00, *p* <0.001), smaller left (*F* (4,482) = 27.38, *p* <0.001) and right HP volume (*F* (4,482) = 33.42, *p* <0.001), more vCSF (*F* (4,482) = 28.94, *p* <0.001) and larger WMH volume (*F* (4,482) = 8.90, *p* <0.001).

Participants who scored above (n = 313) and below (n = 174) a cutoff score of <2 SD on one memory test were compared in a third MANCOVA. Trails B/A ratio violated Levene’s test of equality of error variances, but again inspection of the data showed highly similar variances between the above-cutoff (0.010) and below-cutoff (0.016) groups. Parametric analyses were thus retained. Individuals with episodic memory scores below the cutoff had poorer performance on Category fluency (*F* (4,482) = 11.61, *p* <0.001), BNT (*F* (4,482) = 19.23, *p* <0.001), and Trails B/A ratio (*F* (4,482) = 3.40, *p* = 0.009). They also had smaller BPF (*F* (4,482) = 45.07, *p* <0.001), smaller left (*F* (4,482) = 31.79, *p* <0.001) and right HP volume (*F* (4,482) = 35.16, *p* <0.001), more vCSF (*F* (4,482) = 28.72, *p* <0.001) and larger WMH volume (*F* (4,482) = 9.33, *p* <0.001).

## Discussion

This study aimed to assess how various cognitive, neuroimaging and genetic measures collected at baseline can be used to predict the development of probable AD dementia at 24 months in a sample of elderly participants obtained from ADNI. By assessing a series of normative cutoff scores from cognitive test results, the number of episodic memory and non-memory tests used to assess cognitive performance, and other commonly used neuroimaging and genetic biomarkers, a set of recommended criteria was established which may be used in future investigations to improve prediction for the development of probable AD in the elderly.

Consistent with our initial hypotheses, performance < −1 SD on two memory tests (LM-II and AVLT delay) had the best trade-off between sensitivity and specificity for predicting probable AD, followed by performance < −1.5 SD and < −2 SD on one memory test (LM-II). These results suggest that to maximize diagnostic certainty, a minimum of two measures should ideally be used to assess episodic memory performance and impairment should be defined as scores at least 1 SD below appropriate normative references on both measures. Jak and colleagues [28] were among the first to recommended establishing impairment on at least two measures within a cognitive domain as the best way to increase sensitivity while maintaining reliability, and other authors have since corroborated the value of this approach [6, 29–31]. Our results further indicate that clinicians or researchers with limited resources who administer only a single memory test should opt for a much more stringent cutoff (i.e., −2 SD below normative reference data) to determine episodic memory impairment with comparable accuracy to two measures. Applying a −1.5 SD cutoff to a single test should be avoided when possible, as it remains highly prone to false positive diagnostic errors (c.f. [30, 31]) which reached nearly one-third of the sample (32.6 %) in the present study.

The only variable that improved prediction above and beyond episodic memory testing using two measures was APOE status, consistent with previous research recognizing APOE ε4-positive status as a major risk factor for subsequent AD (see [32] for a review). When only one test was used to assess episodic memory, prediction of dementia was improved using a non-memory test, specifically the ratio of Trails B/A, considered to be a measure of executive control [18]. Predictive accuracy was further increased using APOE ε4 status and whole-brain atrophy (as indexed by brain parenchymal fraction). These interesting results suggest that thorough episodic memory testing using several measures is successful in predicting subsequent dementia with at least as much accuracy as using one memory test plus additional memory tests and biomarkers. It has previously been reported that the use of sensitive neuropsychological instruments are at least as effective in predicting AD as imaging biomarkers [33–36]. Other authors have also reported that the use of a single memory test is not optimal in predicting AD, and that adding information on brain atrophy and/or cerebrospinal fluid biomarkers is necessary to improve predictive accuracy in regression models [35, 37, 38]. We corroborate these findings, and extend them to specify that “impairment” should be defined as performance more than 1 SD below normative data.

Certain limitations must be considered in interpreting these data. First, the ADNI study specifically set out to recruit patients who represented relatively pure cases of MCI and dementia of the Alzheimer’s type, who are appropriate for clinical trials; this is evident in patients’ relatively low burden of WMH [39] (thought to reflect underlying vascular disease [40]). As such, the sample primarily includes individuals whose suspected etiology is AD, and whose primary (and often only) cognitive deficit involves memory. While ADNI provides a large and rich database to study individuals who are at high risk of developing AD, findings generated from these data have limited generalizability to real-world patient populations [39]. Other, more inclusive cohorts of individuals with MCI are needed. In addition, the standardized scores used in this study were derived from published age-adjusted norms for each test. It is possible that the use of local norms may produce different results (e.g., see [41]).

We have shown that diagnostic accuracy can be improved by approximately 10 % by administering an extra memory test to evaluate memory capacities in persons suspected of MCI. This improved accuracy is mostly the result of reducing false positive results, which other authors have shown are inflated when using a single test [31]. Although adding a test to the diagnostic battery resulted in some patients being missed at baseline, who went on to develop AD at 24 months, our findings suggest that this trade-off is altogether fair. An incorrect diagnosis of AD has serious implications for research and clinical practice. First, studies that employ only LM-II to test for memory impairment in participants are effectively pooling true MCI cases with those who are likely cognitively normal, thus potentially weakening the robustness of the research findings and limiting their generalizability. Clinically, the consequences of an incorrect diagnosis include needless testing, pharmacotherapy, and anxiety incurred by the patient and family. Also, inaccurate diagnosis implies that alternative (potentially reversible) causes of cognitive changes are not being investigated.

In closing, we must acknowledge that expanding cognitive batteries to include an extra memory test has some disadvantages. Namely, more clinician time and additional test materials are required, and research protocols will be slightly lengthened. However, we believe that these caveats are greatly outweighed by the benefit of improved accuracy, and that an additional memory measure should be added to clinical and research cognitive batteries to the extent that it is feasible.

## Conclusions

The findings of our study in the ADNI cohort suggest that neuropsychological testing can predict decline with high accuracy regardless of biomarkers, when memory is assessed using delayed recall of a short story and a word list, using a cutoff of < −1 SD below normative references. This criterion provides the optimal trade-off between specificity and sensitivity for predicting conversion to AD at two years. The increased accuracy that this criterion provides decreases the probability of misdiagnosing a patient and avoids needless testing, pharmacotherapy and anxiety, and provides a high-accuracy, low-cost strategy for identifying individuals at highest risk of dementia. In situations where it is only feasible to administer a single memory test, collecting information on non-memory performance and imaging or genetic biomarkers is necessary to optimize diagnostic accuracy.

## Abbreviations

AD: Alzheimer’s disease
ADNI: Alzheimer’s Disease Neuroimaging Initiative
APOE: apolipoprotein E
AUC: area under the curve
AVLT: Rey auditory verbal learning test
BNT: Boston naming test
BPF: brain parenchymal fraction
CDR: Clinical Dementia Rating
FDA: Food and Drug Administration
GM: gray matter
HP: hippocampal
LM-II: logical memory story a delayed recall
MANCOVA: multivariate analysis of covariance
MCI: mild cognitive impairment due to Alzheimer’s disease
MMSE: Mini-mental state exam
MRI: magnetic resonance imaging
NIA: National Institute on Aging
NIBIB: National Institute of Biomedical Imaging and Bioengineering
NINCDS/ADRDA: National Institute of Neurological and Communicative Disorders and Stroke and the Alzheimer's Disease and Related Disorders Association
PET: positron emission tomography
ROC: receiver operating characteristic
SD: standard deviation
SS: scaled scores
TCV: total cranial vault
vCSF: ventricular cerebrospinal fluid
WM: white matter
WMH: white matter hyperintensity
